# Accuracy and impact of prenatal diagnosis in infants with omphalocele

**DOI:** 10.1007/s00383-018-4265-x

**Published:** 2018-04-10

**Authors:** Peter Conner, Jenny Hammarqvist Vejde, Carmen Mesas Burgos

**Affiliations:** 1 Center for Fetal Medicine, Karolinska University Hospital, Stockholm, Sweden; 2 Department of Pediatric Surgery, S3:03, Karolinska University Hospital, 17176 Stockholm, Sweden

**Keywords:** Omphalocele, Exomphalos, Prenatal, Malformations, Beckwith–Wiedemann syndrome

## Abstract

**Background:**

Associated anomalies in omphalocele are common, but to which extent these anomalies are diagnosed before or after birth is less well documented.

**Aim:**

To investigate the different types of associated anomalies, long-term survival and the extent whether these are diagnosed pre- or postnatally in children with a prenatal diagnosis of omphalocele at a single institution.

**Materials and methods:**

Retrospective review of all pregnancies with omphalocele managed and/or born at our institution between 2006 and 2016.

**Results:**

A total of 42 cases with prenatally diagnosed omphalocele were identified. Of those 14 (31%) decided to terminate the pregnancy (TOP). Of the remaining 28 that continued, 12 were giant omphaloceles. The overall mortality rate was 18, 25% for giant and 12% for non-giant omphaloceles. 64% had associated anomalies. Only 1/3 of these anomalies is diagnosed prenatally.

**Conclusion:**

The rate of associated malformations that are diagnosed postnatally is high, but the majority was malformations with a minor clinical significance or impact on future health. Beckwith–Wiedemann syndrome was present only in cases of non-giant omphalocele in our cohort.

## Introduction

Omphalocele is an abdominal wall defect with a birth prevalence of 1 per 4000 livebirths, but a higher incidence if stillbirths and terminations of pregnancy are also taken into account [1–3]. The high rate of associated anomalies and genetic disorders often leads to a termination of pregnancy [3–6]. A non-isolated fetal omphalocele, with an abnormal karyotype and/or associated malformations, constitutes 67–88% of all pregnancies with omphalocele [3–6]. Cardiac and gastrointestinal anomalies are the most common (40–50%), but a broad spectrum of anomalies has been reported [7–10]. Overall, both numerical and structural chromosome anomalies are known to be present in 30–40% of pregnancies with fetal omphalocele [3, 11].

Overall survival rates for liveborn children with omphalocele depend on the severity of the associated anomalies [12, 13]. Isolated cases with omphalocele usually carry a good prognosis [14–16]. Despite improvements in the quality of prenatal examinations and access to both MRI, high-resolution and 3D ultrasound, a large proportion of the associated anomalies will not be diagnosed until after birth [8, 14, 15], and may influence the outcome of the children.

The aim of this study was to document long-term outcomes of prenatally diagnosed omphalocele, to describe the rate and types of associated anomalies diagnosed before and after birth at a single Institution during a 10-year period, and to investigate how these associated anomalies and genetic syndromes affect long-term survival and morbidity.

## Materials and methods

Medical records, including ultrasound reports, from all pregnancies with omphalocele assessed, monitored and managed at our institution between 2006 and 2016 were reviewed. Both cases where the parents decided to continue the pregnancy following a prenatal diagnosis with complete investigation including full karyotyping and detailed ultrasound examination, as well as those that decided to proceed with a termination of pregnancy (TOP) following the diagnosis of omphalocele, with or without associated anomalies, were included.

Data on patient demographics, prenatal imaging, gestational age at birth or TOP, mode of delivery, surgical management, associated anomalies and timing of diagnosis, time to full enteral nutrition, length of hospital stay (LOS), short- and long-term complications were collected from patients records. Postnatal morbidity was defined as either daily use of medications or frequent follow-up for a chronic condition, and/or impairment of neurocognitive, psychomotor or physical capacity.

Data are presented as absolute values (*n*), frequencies (%), mean ± SD, median and ranks. Patients were divided into giant omphaloceles (≥ 5 cm defect with the major part of the liver herniated) or non-giant omphaloceles, as well as isolated vs non-isolated (with any associated chromosomal or structural anomaly) omphaloceles. Statistical analysis was performed using Fisher’s exact test for categorical variables, and Mann–Whitney test for numerical variables, with *p* values < 0.05 considered significant. All data analysis was conducted using GraphPad Prism 6.0 (La Jolla, CA).

## Results

A total of 42 cases of prenatally diagnosed omphaloceles were identified that had been assessed in the second trimester during the period 2006 to 2016. Of these pregnancies, 33% (14) opted for medical termination and there were three cases of spontaneous termination: one miscarriage at 18 gestational weeks (gw), and two cases of intrauterine fetal demise (IUFD) that occurred at 22 and 24 gw. In the group of terminations, both medical and spontaneous (*n* = 17), 71% were Giant Omphaloceles and 64% had an associated anomaly.

In the remaining 25 patients with a prenatal diagnosis of omphalocele that decided to continue their pregnancy, 40% (10/25) were cases of giant omphaloceles. The mean maternal age was 31.7 years (rank 24–38 years), and the mean gestational age at delivery for live born with omphalocele was 36.2 ± 2.58 SD weeks (median 37.2). There were no differences in gender between the groups. There was one case of twin pregnancy. The follow-up time of live born patients with Omphalocele ranged from 1 month to 11 years. (median follow-up time 44 months). All the 25 live born patients underwent surgical repair. The median age at the time of first surgery was 1 day of life (DOL), and the abdomen was closed at a median age of 2 DOL. In 52% (13/25) of cases primary closure of the abdominal wall defect was possible. Preformed Silo bag was used in 32% (8/25) of cases and a patch (synthetic or biological) was used in 36% (9/25). The median length of hospital stay (LOS) was significantly higher in children with giant omphaloceles as well as in those with associated anomalies (Table 1). The postnatal mortality rate for all cases with omphalocele was 8% (2/25 cases), at 6 weeks and 5 months of age, respectively, one of them constituting a case of giant omphalocele. One infant died due to severe pulmonary hypoplasia and pulmonary hypertension, the other patient died at an external institution.

**Table 1:** Characteristics of the cohort with omphalocele divided into subgroups: isolated vs non-isolated, and giant vs non-giant, pairwise comparisons with Fisher’s and Mann–Whitney’s test (*p* < 0.05)

	All omphalocele	Isolated^b^	Non-isolated	Giant	Non-giant
Prenatally diagnosed omphalocele 2006–2016	42	14 (33%)	28 (67%)	22 (52%)	20 (48%)
Continued pregnancy (*n*/%)	28 (67%)	9 (32%)	19 (68%)	12 (43%)	16 (57%)
TOP^a^ (*n*/%)	14 (33%)	5 (36%)	9 (64%)	10 (71%)	4 (29%)
Live born (*n*/%)	25 (89%)	8 (32%)	17 (68%)	10 (40%)	15 (60%)
Live born survival (*n*/%)	23 (92%)	8 (100%)	15 (88%)	9 (90%)	14 (93%)
Overall survival (%)	82%	^a^89%	79%	75%	88%
Associated structural anomalies^c^ (*n*/%)	15 (54%)			6 (50%)	9 (56%)
Genetic syndromes^c^ (*n*/%)	8 (29%)			1 (8%)	7 (44%)*
Any associated anomaly^c^ (*n*/%)	18 (64%)			7 (58%)	11 (69%)
LOS median (days)		12	27*	47	15*
Time to full enteral nutrition, median (days)		8	10	20	7*

Pairwise comparisons, isolated vs non-isolated, giant vs non-giant, Fisher’s test and Mann–Whitney`s test (*p* < 0.05)*

^a^Termination of pregnancy after prenatal counseling

^b^Truly isolated following prenatal and postnatal assessment

^c^Associated structural anomalies or genetic syndromes in continuing pregnancies (*n* = 28)

In the group of giant omphaloceles the mortality rate was 10% (1/10), whereas the mortality was only 7% (1/15) for non-giant omphaloceles.

### Associated anomalies

Associated anomalies were present in 67% (28/42) of all cases with prenatally diagnosed omphaloceles and in 68% (19/28) of pregnancies with omphalocele intending to continue pregnancy following prenatal evaluation.

Of the cases that decided not to continue the pregnancy (*n* = 14), 64% had an associated anomaly: (1) Beckwith–Wiedemann syndrome (BWS), (2) ventricular septum defects (VSD), (3) Cantrell’s pentalogy, two cases of trisomy 18 and one with congenital scoliosis.

At prenatal assessment, associated anomalies were found in only 32% (9/28) of the cases that continued pregnancy and in the remaining 68% (19/28) the omphalocele was considered to be isolated. However, following postnatal examinations or adding information from necropsy, another 36% (10/28) turned out to have an additional anomaly. In the end, only 32% (9/28) were truly isolated cases by definition. Additional anomalies were also found after birth in five out of the nine patients already diagnosed to have associated malformations before birth.

Putting it all together, 68% (19/28) of the pregnancies intending to continue had associated anomalies. A total of 32 anomalies were present in these 19 patients summarized in Table 2. Overall, only one-third (10/32) of all the associated anomalies were diagnosed prenatally. The rate of associated anomalies was higher in non-giant omphaloceles compared to cases of giant omphaloceles (Table 1). Although isolated cases and non-giant omphaloceles had better survival rates, the differences were not statistically significant in our cohort (Table 1).

**Table 2:** Prenatal (a) and postnatally (b) detected associated anomalies among cases of giant and non-giant omphalocele

(a)		
Prenatal detection of associated anomalies *n* = 9/28 patients *n* = 10 malformations	Giant omphalocele (*n* = 12 patients)	Non-giant omphalocele (*n* = 16 patients)
Cardiovascular and pulmonary		
ASD	1	
VSD		3
Overriding aorta		1
Interrupted IHVC	1	1
Gastro-intestinal		
Esophageal atresia	1	
Gallbladder agenesis		1
Uro-genital		
Unilateral renal agenesis		1

(b)		
Postnatal detection of associated anomalies *n* = 15/28 patients *n* = 22 malformations	Giant omphalocele (*n* = 12 patients)	Non-giant omphalocele (*n* = 16 patients)
Cardiovascular and pulmonary		
Coarctation aorta		1
PPHN and lung hypoplasia	1	2
Gastro-intestinal		
Ileum atresia		1
Ano-rectal malformation		1
Uro-genital		
Hydronephrosis	1	
CNS		
Hydrocephalus		1
Corpus callosum agenesis	1	
Cranio-facial		
Cleft palate		1
Ear anomalies		1
Macroglosia		1
Musculo-skeletal		
Syndactyly		1
Genetic/syndromic disorders		
Beckwith–Wiedemann Syndrome		6
Frynn’s Syndrome		1
Trisomy 18	1	
Cloacal malformation, OEIS	1	

Beckwith–Wiedemann syndrome (BWS) was present in 25% of all children with omphalocele, but was only observed in cases with non-giant omphaloceles. The rate of BWS in cases with non-giant omphalocele was 43%.

Despite the high rate of associated anomalies, more than 70% of surviving children born with omphalocele had non-significant morbidity, with only a minor impact on their quality of life.

## Discussion

Despite extensive prenatal evaluation with ultrasound performed by a fetal medicine specialist at a tertiary level referral center over a 10-year period, many of the infants with a prenatal diagnosed omphalocele turned out to have additional anomalies that were diagnosed first after birth, almost two-thirds (22/32).

Many of the anomalies diagnosed postnatally were considered to be of minor importance, and all patients with associated anomalies diagnosed exclusively at this time survived. However, it is important to be aware of the high rate of associated anomalies that will be diagnosed following birth when counseling parents to pregnancies with a prenatal diagnosis of omphalocele.

In our cohort, 33% opted for TOP after prenatal counseling, which is lower than the numbers stated in general in the Swedish national registry of congenital malformations. This may be due to the fact that almost all parents were counseled by a multidisciplinary team following a prenatal diagnosis, implicating that the expecting parents may receive more precise information and an accurate prognosis than what is usually offered at non-tertiary centers. In general in Sweden, approximately 60% of pregnancies with a prenatal diagnosis of omphalocele are terminated [17]. In the majority of these cases, chromosomal abnormalities or associated major anomalies are present, leaving a selected population of infants born with omphalocele where prenatal diagnosis in the second trimester has assessed the malformation to be isolated. However, the findings in our study indicate that the actual proportion of infants with an isolated omphalocele is low, only 32%. Among the cases that opted for TOP; the rate of associated anomalies did not differ from those who continued their pregnancy, but the anomalies were all severe in this group. Overall, we found a total of 32 associated anomalies among our 19 non-isolated cases of children born with omphalocele. Despite the high numbers of non-isolated cases, most of these children in our cohort live an apparently healthy and normal life.

The expect rum of anomalies described are in accordance with previous reports [8, 9, 18, 19].

Beckwith–Wiedemann syndrome (BWS) was to a very large extent associated with non-giant omphalocele. BWS, is an over-growth disorder characterized by neonatal hypoglycemia, macrosomia, macroglossia, hemihypertrophy, visceromegaly and with a risk to develop embryonal tumors (Wilms tumor, hepatoblastoma, neuroblastoma and rhabdomyosarcoma). The syndrome can be diagnosed through molecular testing, demonstrating either abnormalities involving chromosome 11p15, disturbed methylation or uniparental disomy in approximately 80% of cases [20, 21]. This was the only chromosomal aberration found postnatally among babies born with omphalocele, since those with a prenatal diagnosis of trisomy 13 or 18, had already terminated their pregnancies.

Prenatal investigation of BWS was not routinely offered at our institution in pregnancies with omphalocele until the last year of the study period explaining why we did not have any cases of BWS with a prenatal diagnosis. Clinical signs of BWS, e.g. macrosomia and macroglossia often appear late in the third trimester, a period of pregnancy during which genetic testing and also TOP is not performed or allowed in our setting.

Despite the fact that giant omphalocele constitutes a more severe defect in terms of surgical management, the rate of associated anomalies was lower than in cases with non-giant omphaloceles. The survival rate in the group with giant omphaloceles was lower, but not significantly different to non-giant omphaloceles most likely due to the small number of cases.

Isolated cases of omphalocele have been reported to have better outcomes [14–16]. We did not find any significant differences in terms of survival between the two groups. This could be due to relatively small numbers in our cohort, one of the major limitations of this study.

In our center, the preferred approach is to perform surgery with the aim to try to close the defect, including the larger defects with a staged closure. In other centers a different approach is taken, large omphaloceles are managed conservatively, allowing the sac to epithelialize and postponing surgery till later in life [22].

In conclusion, we believe that the results of this study are important to conceive when counseling parents following a prenatal diagnosis of omphalocele, since many of the non-severe anomalies will not be diagnosed before birth, but at the same time will probably not affect survival. Cases with either severe associated anomalies or chromosomal aberrations differ from non-severe cases both in terms of survival and morbidity deeply affecting the outcome. Moreover, despite the large number of associated anomalies, our long-term data reveal that the majority of children live born with omphalocele suffer no major co-morbidity and will have a good quality of life.

## References

[1] Bugge M, Hauge M (1983). Gastroschisis and omphalocele in Denmark. An epidemiological study. Ugeskrift for laeger.

[2] Tan KB, Tan KH, Chew SK (2008). Gastroschisis and omphalocele in Singapore: a ten-year series from 1993 to 2002. Singap Med J.

[3] Wilson RD, Johnson MP (2004). Congenital abdominal wall defects: an update. Fetal Diagn Ther.

[4] Gilbert WM, Nicolaides KH (1987). Fetal omphalocele: associated malformations and chromosomal defects. Obstet Gynecol.

[5] Greenwood RD, Rosenthal A, Nadas AS (1974). Cardiovascular malformations associated with omphalocele. J Pediatr.

[6] Stoll C, Alembik Y, Dott B (2008). Omphalocele and gastroschisis and associated malformations. Am J Med Genet Part A.

[7] Hughes MD, Nyberg DA, Mack LA (1989). Fetal omphalocele: prenatal US detection of concurrent anomalies and other predictors of outcome. Radiology.

[8] Corey KM, Hornik CP, Laughon MM (2014). Frequency of anomalies and hospital outcomes in infants with gastroschisis and omphalocele. Early Human Dev.

[9] Benjamin B, Wilson GN (2014). Anomalies associated with gastroschisis and omphalocele: analysis of 2825 cases from the Texas Birth Defects Registry. J Pediatr Surg.

[10] Ani MA, Khan SA (2014). Omphalocele with intra abdominal anomalies. J Neonat Surg.

[11] St-Vil D, Shaw KS, Lallier M (1996). Chromosomal anomalies in newborns with omphalocele. J Pediatr Surg.

[12] Brantberg A, Blaas HG, Haugen SE (2005). Characteristics and outcome of 90 cases of fetal omphalocele. Ultrasound Obstet Gynecol.

[13] Marshall J, Salemi JL, Tanner JP (2015). Prevalence, correlates, and outcomes of omphalocele in the United States, 1995–2005. Obstet Gynecol.

[14] Cohen-Overbeek TE, Tong WH, Hatzmann TR (2010). Omphalocele: comparison of outcome following prenatal or postnatal diagnosis. Ultrasound Obstet Gynecol.

[15] Porter A, Benson CB, Hawley P (2009). Outcome of fetuses with a prenatal ultrasound diagnosis of isolated omphalocele. Prenatal Diagn.

[16] Heider AL, Strauss RA, Kuller JA (2004). Omphalocele: clinical outcomes in cases with normal karyotypes. Am J Obstet Gynecol.

[17] Birth Defects (2014) The Swedish National Board of Health and Welfare. http://www.socialstyrelsen.se/publikationer2016/2016-3-4. Captation 2016-11-21

[18] Blazer S, Zimmer EZ, Gover A (2004). Fetal omphalocele detected early in pregnancy: associated anomalies and outcomes. Radiology.

[19] von Oeyen P, Holmes LB, Trelstad RL (1982). Omphalocele and multiple severe congenital anomalies associated with osteodysplasty (Melnick–Needles syndrome). Am J Med Genet.

[20] Wilkins-Haug L, Porter A, Hawley P (2009). Isolated fetal omphalocele, Beckwith–Wiedemann syndrome, and assisted reproductive technologies. Birth Defects Res Part A Clin Mol Teratol.

[21] Chen CP (2007). Syndromes and disorders associated with omphalocele (I): Beckwith–Wiedemann syndrome. Taiwan J Obstet Gynecol.

[22] Ledbetter DJ (2012). Congenital abdominal wall defects and reconstruction in pediatric surgery: gastroschisis and omphalocele. Surg Clin N Am.

